# Exome sequencing resolves apparent incidental findings and reveals further complexity of SH3TC2 variant alleles causing Charcot-Marie-Tooth neuropathy

**DOI:** 10.1186/gm461

**Published:** 2013-06-27

**Authors:** James R Lupski, Claudia Gonzaga-Jauregui, Yaping Yang, Matthew N Bainbridge, Shalini Jhangiani, Christian J Buhay, Christie L Kovar, Min Wang, Alicia C Hawes, Jeffrey G Reid, Christine Eng, Donna M Muzny, Richard A Gibbs

**Affiliations:** 1 Department of Molecular and Human Genetics, Baylor College of Medicine, One Baylor Plaza, Houston, TX 77030, USA; 2 Human Genome Sequencing Center, Baylor College of Medicine, One Baylor Plaza, Houston, TX 77030, USA; 3 Whole Genome Laboratory, Baylor College of Medicine, One Baylor Plaza, MS: NAB2015, Houston, TX 77030, USA

**Keywords:** Exome sequencing, Whole-genome sequencing, Incidental findings, SH3TC2, Personal genomes, Precision medicine

## Abstract

**Background:**

The debate regarding the relative merits of whole genome sequencing (WGS) versus exome sequencing (ES) centers around comparative cost, average depth of coverage for each interrogated base, and their relative efficiency in the identification of medically actionable variants from the myriad of variants identified by each approach. Nevertheless, few genomes have been subjected to both WGS and ES, using multiple next generation sequencing platforms. In addition, no personal genome has been so extensively analyzed using DNA derived from peripheral blood as opposed to DNA from transformed cell lines that may either accumulate mutations during propagation or clonally expand mosaic variants during cell transformation and propagation.

**Methods:**

We investigated a genome that was studied previously by SOLiD chemistry using both ES and WGS, and now perform six independent ES assays (Illumina GAII (x2), Illumina HiSeq (x2), Life Technologies' Personal Genome Machine (PGM) and Proton), and one additional WGS (Illumina HiSeq).

**Results:**

We compared the variants identified by the different methods and provide insights into the differences among variants identified between ES runs in the same technology platform and among different sequencing technologies. We resolved the true genotypes of medically actionable variants identified in the proband through orthogonal experimental approaches. Furthermore, ES identified an additional SH3TC2 variant (p.M1?) that likely contributes to the phenotype in the proband.

**Conclusions:**

ES identified additional medically actionable variant calls and helped resolve ambiguous single nucleotide variants (SNV) documenting the power of increased depth of coverage of the captured targeted regions. Comparative analyses of WGS and ES reveal that pseudogenes and segmental duplications may explain some instances of apparent disease mutations in unaffected individuals.

## Background

Exome sequencing (ES) is an approach to human genome analysis and genetic variant detection that focuses on the coding exons of genes and closely linked functional elements. ES is less expensive than comparable approaches based on whole genome sequencing (WGS), because there is overall less raw DNA sequence data required. Furthermore, coding regions contain the changes that are currently the most easily interpretable, and knowledge gained from ES may therefore have more immediate medical utility and application. There is a generally held belief, however, that WGS may be more informative than ES, even within the boundaries of exome regions, as the random distribution of individual sequence reads offers an overall higher likelihood of effective testing of individual sites in the genome. Further, biases inherent to the capture process may result in missing or poorly covered bases in a subset of the exome regions [1].

We previously applied WGS using massively parallel next-generation sequencing (NGS) methods to a subject with molecularly undefined, autosomal recessive, Charcot-Marie-Tooth (CMT) neuropathy and identified apparently causative compound heterozygous *SH3TC2 *variants: nonsense p.R954X (g. chr5: 148,406,435 G>A) and missense p.Y169H alleles (g. chr5: 148,422,281 A>G) [2]. The two alleles satisfied multiple criteria for disease causation: both were validated with alternate technologies; the nonsense p.R954X allele had been previously reported in unrelated CMT families and corroborated with functional studies; each segregated faithfully in the pedigree and no other lesion was discovered in the locus despite adequate coverage of all coding bases with NGS reads. Further, each allele faithfully independently segregated with additional different mild phenotypes observed in other family members - susceptibility to carpal tunnel syndrome (presumably due to loss-of-function nonsense mutation) and an electrophysiologically identified subclinical axonopathy (missense allele).

**Figure 1:**
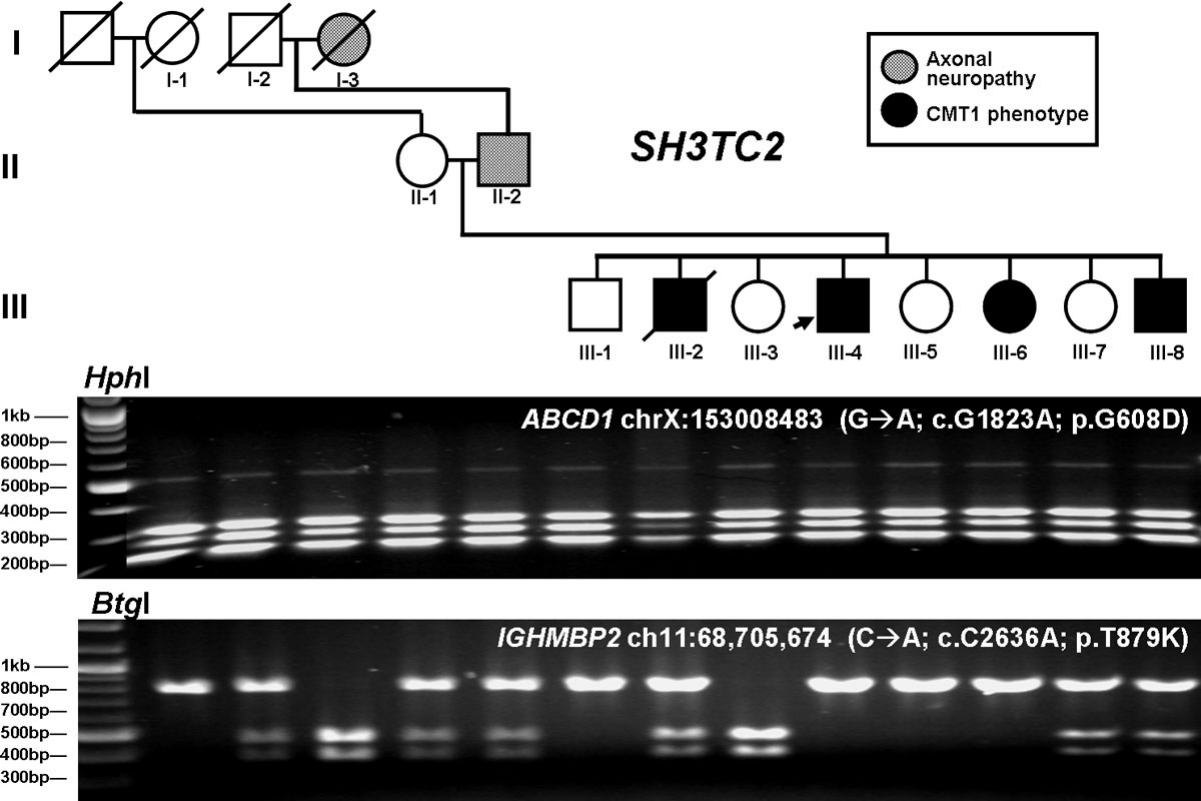
**Confirmation and segregation by endonuclease restriction digestion of mutations presumed to represent incidental findings**. The upper gel shows restriction digestion using the *Hph*I restriction enzyme for the p.G608D mutation in *ABCD1*, causative for adrenoleukodystrophy (ALD). All tested individuals, none of which presented with ALD, are shown to be heterozygous for the mutation, including all males whom are hemizygous for genes on the × chromosome. The lower gel shows restriction digestion using the *Btg*I restriction enzyme for the p.T879K mutation in *IGHMBP2*, putatively causative for spinal muscular atrophy with respiratory distress type 1 (SMARD1). Random segregation and zygosity for this variant can be observed in this family; none of the individual subjects present with SMARD.

Unresolved issues in the study included discovery of additional reportedly pathogenic alleles at other loci, related to potentially medically actionable conditions (incidental or secondary findings). For example, the proband was suggested to have a dominant variant that was causative for adrenoleukodystrophy (ALD, MIM #300100) [3]; *ABCD1*, chrX:153,008,483 G>A; p.G608D, as well as to be homozygous for an allele causative for spinal muscular atrophy with respiratory distress type 1 (SMARD, MIM #604320) [4]; *IGHMBP2*, chr11:68,705,674C>A; pT879K. These variants were confirmed by Sanger sequencing and restriction endonuclease digestion (Figure 1). The proband was also found by WGS to be homozygous for reported causative mutations in three other Mendelian disease genes. The possibility for erroneous entries in public databases and the lack of clinical indications for any of these five conditions, led to the conclusion that these alleles were most probably not pathogenic in the proband.

In the period since the initial CMT study, technologies for both WGS and ES have advanced considerably. We developed a series of improved ES methods and reagents that reliably assay >95% of the coding regions of the human genome [5,6]. The current ES assay design involves routine 'oversampling' of the targeted regions, and ensures that a minimum of 95% (average 97%) of these positions are tested with >20-fold NGS coverage. The new methods also take advantage of longer individual DNA sequence reads generated on the Illumina HiSeq platform (2 × 100 bp) *versus *the previous SOLiD platform (2 × 50 bp) used for WGS.

## Methods

### Sample inclusion

This study conforms to the Helsinki Declaration regarding ethical principles for medical research involving human subjects. Subject BAB195 and additional family members were recruited and consented for genomic and DNA studies under a research protocol approved by the Institutional Review Board of Baylor College of Medicine. All patients gave informed consent for participation in this study.

### DNA preparation methods

Blood was directly collected in PAXgene Blood DNA tubes and DNA was isolated using the PAXgene Blood DNA kit (PreAnalytiX, Qiagen, Valencia, CA, USA). The quality of the DNA sample was ascertained by electrophoresis and determined to be of high quality (size >23 kb) with no visible degradation. Quantity was determined using standard Pico Green assays.

### Description of NimbleGen VCRome 2.1 exome capture design

In the current methods, the HGSC-design 'VCRome 2.1' reagent [7] was used for exome capture. This NimbleGen probe set targets the Vega [8], CCDS, and RefSeq gene models, including predicted genes within RefSeq, as well as microRNA (miRNA) [9] and regulatory regions for a total target size of 42 Mb. Thus, exome capture sequencing will identify SNPs and indels in protein coding regions, exon flanking sequences (including intron splice sites), regulatory regions, and small non-coding RNAs (for example, microRNAs).

### Illumina exome sequencing on GAII and HiSeq

#### Library construction

Genomic DNA samples were constructed into Illumina PairEnd pre-capture libraries according to the manufacturer's protocol (Illumina Multiplexing_SamplePrep_Guide_1005361_D) with modifications as described in the *BCM-HGSC Illumina Barcoded Paired-End Capture Library Preparation *protocol. The complete library and capture protocol, as well as oligonucleotide sequences are accessible from the HGSC website [10].

Briefly, 1 ug of genomic DNA in 100 uL volume was sheared into fragments of approximately 300 base pairs in a Covaris microTube with the E2 system (Covaris, Inc., Woburn, MA, USA) followed by end-repair, A-tailing, and ligation of the Illumina multiplexing PE adaptors. Pre-capture Ligation Mediated-PCR (LM-PCR) was performed for seven cycles of amplification using the 2X SOLiD Library High Fidelity Amplification Mix (custom product manufactured by Invitrogen). Universal primer IMUX-P1.0 and pre-capture barcoded primer IBC were used in the PCR amplification. Purification was performed with Agencourt AMPure XP beads. Quantification and size distribution of the pre-capture LM-PCR product was determined using the Agilent Bioanalyzer 2100 DNA 7500 chip.

#### Capture methods

Pre-capture libraries (1 ug) were hybridized in solution to the VCRome 2.1 exome capture platform (HGSC design, NimbleGen) described above according to the manufacturer's protocol *NimbleGen SeqCap EZ Exome Library SR User's Guide (Version 2.2) *with minor revisions. Human CotI DNA and full-length Illumina adaptor-specific blocking oligonucleotides were added into the hybridization mix to block repetitive genomic sequences and the adaptor sequences. Post-capture LM-PCR amplification was performed using the 2X SOLiD Library High Fidelity Amplification Mix with 14 cycles of amplification. After the final XP bead purification, quantity and size of the capture library was analyzed using the Agilent Bioanalyzer 2100 DNA Chip 7500. The efficiency of the capture was evaluated by performing a qPCR-based quality check on the four standard NimbleGen internal controls. Successful enrichment of the capture libraries was estimated to be in the range of 7 to 9 of ΔCt value over the non-enriched samples.

#### Illumina sequencing on GAIIx and HiSeq 2000

Library templates were prepared for sequencing using Illumina's cBot cluster generation system with the corresponding TruSeq PE Cluster Kits for the GA and HiSeq. Briefly, these libraries were denatured with sodium hydroxide and diluted to 3 to 6 pM in hybridization buffer in order to achieve a load density of 400 to 550 k clusters/mm^2 ^on the GAIIx and 700 to 900 k clusters/mm^2 ^on the HiSeq 2000. Barcoded libraries were loaded in two independent lanes of a GAIIx flow cell and then merged for analysis. Due to the higher capacity of the HiSeq 2000, each barcoded sample was pooled (in a set of three) for loading onto a single lane of a HiSeq flowcell and then deconvoluted for analysis based on barcode sequence. All lanes were spiked with 2% phage phiX DNA control library for run quality control. After loading onto the flow cell, sample libraries underwent bridge amplification to form clonal clusters, and the sequencing primer was hybridized.

Sequencing runs were performed in paired-end mode using the Illumina Genome Analyzer IIx (GAIIx) and HiSeq 2000 platforms. Using the TruSeq SBS Kits for the GA and the HiSeq, sequencing-by-synthesis reactions were extended for 101 cycles from each end, with an additional seven cycles for the index read for runs that included pooled samples. Real-time analysis (RTA) software was used to process the image analysis and base calling. Sequencing runs generated approximately 40-65 million and 350-500 million successful reads per lane for the GAIIx and HiSeq, respectively. With these run yields, each sample library achieved 10 to 12 Gb of raw DNA sequence data, which enabled a minimum of 20x coverage for 95% to 97% of the bases targeted in the exome.

### Illumina whole genome sequencing on HiSeq 2000

The WGS library was sequenced with the Illumina HiSeq 2000 platform using the template preparation and sequencing methods described above for the HiSeq 2000, with the following exceptions: a total of three flow cell lanes were loaded, yielding approximately 148 Gb of sequence for the WGS dataset.

### Illumina analysis and SNP calls

Illumina sequence analysis was performed using the HGSC Mercury analysis pipeline [11] that manages all aspects of data processing and analyses, moving data step by step through various analysis tools from the initial sequence generation on the instrument to annotated variant calls (SNPs and intra-read in/dels). First, the primary analysis software on the instrument produces .bcl files that are transferred off-instrument into the HGSC analysis infrastructure by the HiSeq Real-time Analysis module (1.13.48). Once the run is complete and all .bcl files are transferred, Mercury runs the vendor's primary analysis software (CASAVA v1.8.0), which de-multiplexes pooled samples and generates sequence reads and base-call confidence values (qualities). The next step is the mapping of reads and qualities to the GRCh37 human reference genome assembly [12] using the Burrows-Wheeler aligner (BWA [13,14]) and producing a BAM [15] (binary alignment/map) file. The third step involves quality recalibration (using GATK [16,17]), and where necessary the merging of separate sequence-event BAMs into a single sample-level BAM is performed. BAM sorting, duplicate read marking, and realignment to improve in/del discovery all occur at this step. Next, we used the Atlas2 [18] suite (Atlas-SNP and Atlas-indel) to call variants and produce a variant call file (VCF [19]). Finally, annotation data is added to the vcf using the Cassandra [20] suite of annotation tools that brings together relevant annotation information using ANNOVAR [21] with UCSC and RefSeq gene models, as well as a host of other internal and external data resources.

### Ion torrent exome sequencing using PGM and Proton

***Library construction: ***The pre-capture libraries for the Ion Torrent platforms (PGM and Proton) were constructed using the Ion Xpress Plus Fragment Library Kit (Life Technologies) and the experimental procedures followed the manufacturer's Ion Xpress Plus gDNA and Amplicon Library Preparation User Guide with minor modifications.

The PGM pre-capture library was generated using 1 ug of genomic DNA which was fragmented to approximately 200 base pairs by the Covaris S2 system (Covaris, Inc., Woburn, MA, USA) and purified with 1.2x Agencourt Ampure XP Beads (Beckman Coulter, Cat. No. A63882). Fragmentation was followed by end-repair, blunt-end ligation of the Ion Xpress Barcode and Ion P1 adaptors as well as nick translation. Pre-capture ligation-mediated PCR (LM-PCR) was performed for eight cycles of amplification using 2X SOLiD Library High Fidelity Amplification Mix (custom product by Invitrogen).

The Proton pre-capture library was generated in a similar manner as above starting with fragmentation of genomic DNA to 100 base pairs using the Covaris S2 system and purified with 1.8x Agencourt Ampure XP Beads. Blunt-end ligation was performed using the non-barcoded Ion A and Ion P1 adaptors. Post-ligation size selection was performed using a 3% agarose gel cassette with the target size of 180 bp. Final pre-capture LM-PCR was performed again using 2X SOLiD Library High Fidelity Amplification Mix (custom product by Invitrogen), for 12 cycles of amplification. The above enzymatic reactions for both the ION Torrent PGM and Proton libraries were followed by AMPure XP bead purification (1.4x for the PGM library and 1.8x for the Proton library, respectively). Quantity and size distribution of the PCR product were analyzed using the Agilent Bioanalyzer 2100 DNA 7500 chip.

***Capture methods: ***For target enrichment, 1 ug of each pre-capture library was hybridized in solution to the previously described HGSC VCRome 2.1 exome capture design following the Manual of Ion TargetSeq Custom Enrichment Kits with minor modifications. Human CotI DNA and adaptor A and P1-specific blocking oligonucleotides were added into the hybridization reactions to block repetitive genomic sequences and the common adaptor sequences. Post-capture LM-PCR amplification was performed using 2X SOLiD Library High Fidelity Amplification Mix with 14 to 16 cycles of amplification. Quantities and sizes of the capture libraries were analyzed using the Agilent Bioanalyzer 2100 DNA Chip 7500. The efficiency of the capture was again evaluated by performing a qPCR-based quality check on the four standard NimbleGen internal controls. Successful enrichment of the capture libraries was estimated to range from a 7 to 9 of ΔCt value over the non-enriched samples.

***Template preparation and sequencing: ***Library templates were prepared for sequencing using the Life Technologies Ion Xpress and Ion OneTouch protocols and reagents. Briefly, library fragments were clonally amplified onto ion sphere particles (ISPs) through emulsion PCR and then enriched for template-positive ISPs. More specifically, PGM emulsion PCR reactions utilized the Ion Xpress 200 Template Kit (Life Technologies) as specified by the vendor. Emulsions were generated with an IKA Ultra-Turrax, amplification followed using standard thermal cycling methods, and the ISPs were recovered with a SOLiD emulsion collection tray (Life Technologies) through centrifugation. In some instances, these amplification and recovery steps were automated for the PGM reactions using the Ion OneTouch System and the Ion OneTouch Template Kit v2, and similarly, the Proton emulsion PCR reactions were performed using the Ion Proton I Template OT2 Kit (Life Technologies), with amplification and recovery steps automated with the Ion OneTouch 2 System. Following recovery, enrichment was completed by selectively binding the ISPs containing amplified library fragments to streptavidin coated magnetic beads, removing empty ISPs through washing steps, and denaturing the library strands to allow for collection of the template-positive ISPs. For all reactions, these steps were accomplished using the Life Technologies ES module of the Ion OneTouch 2 System, and template-positive ISPs were quantified using the Guava EasyCyte 5 (Millipore Technologies), obtaining >90% enrichment efficiency for all reactions.

For PGM runs, approximately 35 million template-positive ISPs per run were deposited onto the Ion 318C chips (Life Technologies) by a series of centrifugation steps that incorporated alternating the chip directionality. Sequencing was performed with the Ion PGM 200 Sequencing Kit (Life Technologies) using the 440 flow ('200 bp') run format. For the single Proton run, the Proton P1 Chip was first pre-rinsed and incubated with NaOH for 1 min before loading in order to minimize residual contaminants and decrease background signal. Approximately 300-350 million template-positive ISPs were deposited onto the Proton PI Chip and then underwent sequencing using the Proton's 260 flow ('100 bp') run format and the Life Technologies Ion Proton I Sequencing Kit. A total of nine PGM runs and one Proton run were sequenced, which yielded a total of 6.2 and 7.9 Gb of data, respectively. Analyses showed that 91% to 92% of the targeted exome bases were covered to a depth of at least 20x.

***Ion Torrent analysis methods: ***Sequence data from either Ion Torrent Personal Genome Machine (PGM) or Proton were processed on the instrument server using the Torrent Suite v3.2 software package. These include signal processing, base calling, and mapping. Reads were mapped to the GRCh37 human reference genome assembly [22] via TMAP short read aligner [23]. In the case of the Proton platform, reads identified with the same start coordinate and 3' adaptor flow position were considered duplicates and were removed via a proprietary method in the Torrent Suite. For the PGM, duplicate reads were identified solely by start position and were removed using the Picard MarkDuplicates tool [24]. Where multiple sequencing runs were generated on the same library, BAM files were combined into a single BAM file with Picard MergeSamFiles prior to identifying and removing duplicates.

Single nucleotide variants (SNVs) were identified using VarIONt, an extensible and highly configurable variant caller pipeline developed at the HGSC and specifically tuned for Ion Torrent sequence. It utilizes SAMtools pileup [25] to generate a pileup string at every mapped base coordinate. It filters out portions of the read that may be unreliable, and then identifies alternate alleles from the remainder. Part of VarIONt's capability is to define thresholds at which variants are called. For this application, we required that the interrogation site have a minimum read coverage of 10X and a minor allele fraction (maf) of 0.05. High confidence variants must have a minimum read depth of 30X and 0.1 maf. Once the variants were called, the previously described Cassandra annotation suite was used to annotate the identified variants.

### SOLiD whole genome sequencing

SOLiD data used for comparative analyses, referred to here as SOLiD WGS, were originally obtained as specified in Lupski *et al. *[2]. In summary, whole genome sequence data were generated at 29.6x average depth of coverage using the Sequencing by Oligonucleotide Ligation and Detection (SOLiD) technology (Life Technologies, formerly Applied Biosystems), with a mappable yield of 89.6 Gb. Mapping and variant calling analyses were performed using the analysis suite Corona Lite.

### Data release

This study has been registered at the National Center for Biotechnology Information (NCBI) under BioProject ID 203659 (Accession: PRJNA203659) for BioSample: SAMN00009513. Data are publicly available through the Sequence Read Archive (SRA) under accession numbers: SRX286243, SRX286245, SRX286282, SRX286417, SRX286419, SRX286832.

### Data analysis

Data analyses and comparison of variants between the multiple sequencing runs were performed using custom Perl scripts.

### Confirmation of variants

Variants of interest were confirmed by Sanger sequencing of amplified PCR products. Primers specific to the region containing the variant to be tested were designed. Standard end-point PCR was performed using QIAGEN HotStar *Taq *polymerase (QIAGEN Sciences, Maryland, USA). For *ABCD1 *fragment amplification, long-range PCR was performed using the QIAGEN Long-range PCR mix. Amplification of specific PCR fragments was confirmed by agarose gel electrophoresis. Endonuclease restriction digestion was performed to orthogonally test the genotype and segregation of some of the variants of interest. The amplified PCR products were digested using specific restriction enzymes (New England BioLabs) to identify the site of the mutations to be tested. Specific endonuclease digestion was verified by agarose gel electrophoresis.

## Results and Discussion

To pursue unresolved issues from the previous WGS study that included unconfirmed variant calls and the challenging interpretation of incidental variants, we further tested the subject's DNA with the latest version of ES methods. A total of almost 60 Gb of new raw data, with an average of 11 Gb per exome sequence run, were generated. An average depth of sequence coverage of 170 and a median coverage of 140 was attained for the main captured exome run (HiSeq-1). More than 97% of the targeted DNA bases were covered with >20 reads and >94% were covered with >40 reads (Table 1). For exome enrichment and capture we used the in-house designed VCRome 2.1 exome targeting reagent that covers 197,583 regions with a total number of 35,432,211 bases targeted. A median of 91,573 SNPs were recovered by the different ES experiments, however not all of the SNPs recovered per exome run are coding exonic variants.

**Table 1:** SNV identified by replicated ES of CMT proband.

Platform	GAII-1	GAII-2	HiSeq-1	HiSeq-2	PGM	Proton	WGS-SOLID	WGS-HiSeq
Total reads produced	117,685,348	107,207,362	119,508,046	118,716,380	41,268,598	71,113,537	2,469,936,140	1,479,177,846

Duplicate reads (%)	4.98	3.63	5.86	5.74	25.10	19.34	5.00	2.29

Total reads aligned (%)	97.80	98.66	96.76	97.09	99.16	98.83	58.12	96.57

Aligned reads on target (%)	64.90	69.70	70.59	70.66	60.81	71.66	N/A	N/A

Average coverage	138x	137x	170x	169x	65x	93x	29.6x	47.6x

Median coverage	110x	111x	140x	139x	60x	80x	N/A	40x

Targets hit (%)	99.42	99.32	99.42	99.40	99.22	99.29	N/A	N/A

Bases targeted	35,432,211	35,432,211	35,432,211	35,432,211	35,432,211	35,432,211	N/A	N/A

Targeted bases with 10+ coverage (%)	97.58	97.49	97.98	97.99	95.84	94.64	N/A	N/A

Targeted bases with 20+ coverage (%)	96.19	96.18	97.23	97.21	92.28	91.28	N/A	N/A

Targeted bases with 40+ coverage (%)	90.60	91.08	94.59	94.53	75.19	80.75	N/A	N/A

Total SNPs	102,444	91,661	92,553	91,484	41,958	57,243	3,420,306	4,016,486

cSNPs (*n*)	23,321	22,869	23,017	22,980	20,372	22,079	18,829	24,475

nsSNPs (*n*)	11,491	11,217	11,288	11,252	11,363	10,821	9,069	12,370

Total c_inDels	8,879	8,249	9,576	9,375	N/A	N/A	N/A	10,439

**Figure 2:**
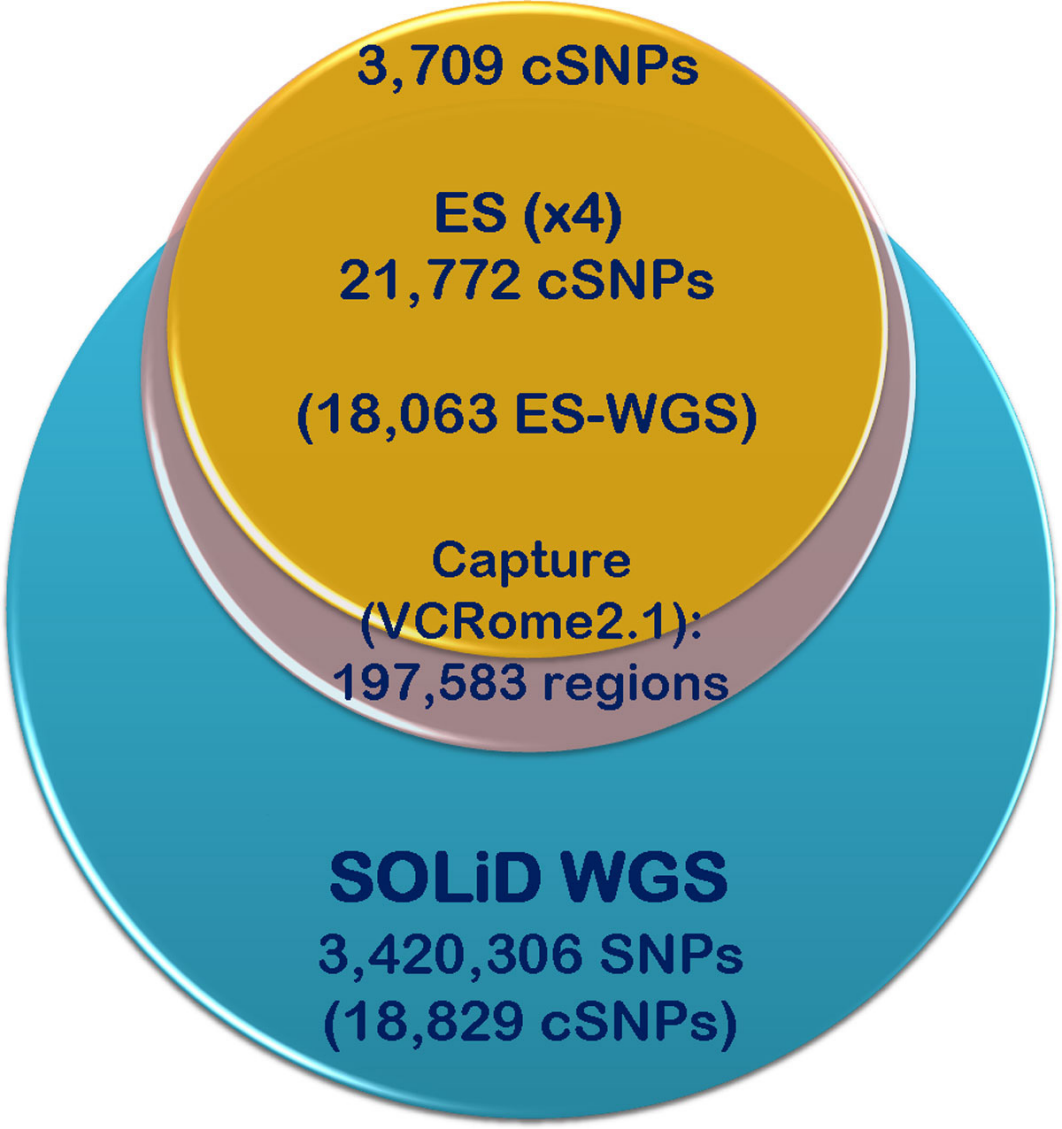
**Diagram depicting the differences between the number of variants identified by WGS and ES of the same individual genome**. WGS identified 3,420,306 SNPs throughout the genome, including 18,829 coding SNPs (cSNPs). Targeted exome sequence (ES) focuses on capturing most of the coding variation contained in 197,583 exonic regions (VCRome 2.1 design). From this, ES identified 21,772 concordant cSNPs among four Illumina sequencing runs and 18,063 high-quality cSNP variants concordant among all six exome sequencing experiments. Of these, 3,709 cSNPs difered from the cSNPs identified by the original SOLiD WGS approach.

**Figure 3:**
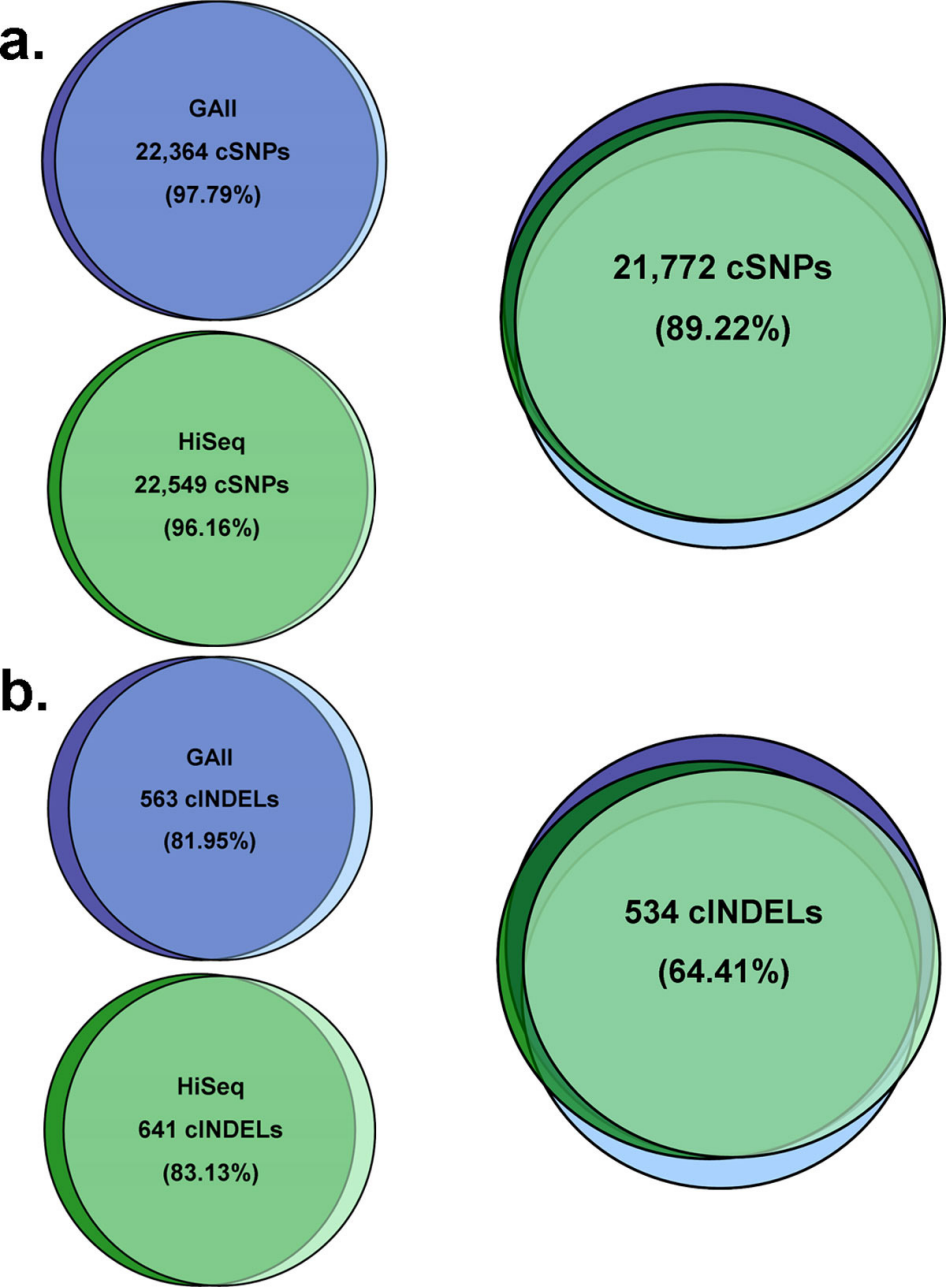
**Overlap of SNVs identified in four targeted exome sequencing replicates of the same individual's DNA in the Illumina platform**. (**a**) Comparison of identified coding SNPs (cSNPs) within and between sequencing technologies (GAII *vs*. HiSeq). There is a high percentage of shared identified SNPs both within and between the different technologies. (**b**) Comparison of identified InDels within and between sequencing technologies. We observe less overlap between the two technologies probably due to a higher rate of false positive InDels.

The ES data recovered a larger number of variants in the coding regions than was seen by WGS (Figure 2), suggesting a higher sensitivity, but also raising the possibility of a higher false positive rate. The number of ES variants was similar in four experiments using the same chemistry (Illumina GAII (two runs) *vs*. Illumina HiSeq (two runs), Table 1, Figure 3). For coding SNPs, the intra-platform concordance between pairs of ES experiments ranged from 96.16% to 97.79%, while the concordance among the four ES experiments performed using the reversible terminators chemistry was 89.22% (Figure 3a). Concordance of indel variants between runs in the same platform ranged from 81.95% to 83.13%; however this decreases when comparing across the four Illumina experiments (64.41%) (Figure 3b), which suggests that some of the indel calls can be platform-specific artifacts. Indels are in general more difficult to assess by variant callers than single-nucleotide substitutions and consequently some of the differences and the lower concordance for indel calling within and between platforms can also arise at the alignment and variant calling stage of the data processing.

**Figure 4:**
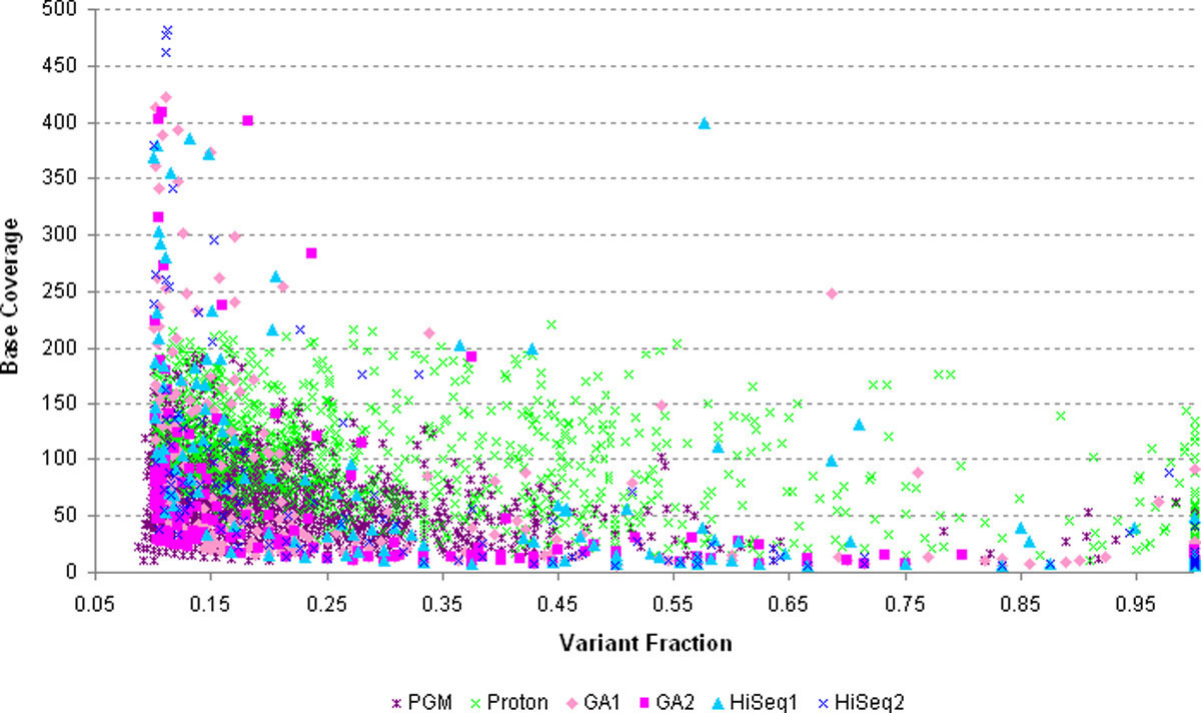
**Distribution of variant fraction *vs***. coverage per base for SNPs called only in single exome sequencing runs, not replicated in any other exome run. For the six different exome sequencing runs, the majority of single-run called SNPs fall below the 0.20-0.25 variant fraction for reliable heterozygous calls; those single-run calls with higher variant fractions (>0.20) generally fall below the 10x coverage cutoff. The run that had the most 'private' calls was the PGM run; however the majority of these private calls fall within the low variant fraction portion of the distribution suggesting that most of them are false positives. Interestingly, the distribution of 'private' SNPs in the Proton exome sequencing run is more scattered and distributed in the coverage and variant fraction ranges usually observed for true positive variant calls.

**Figure 5:**
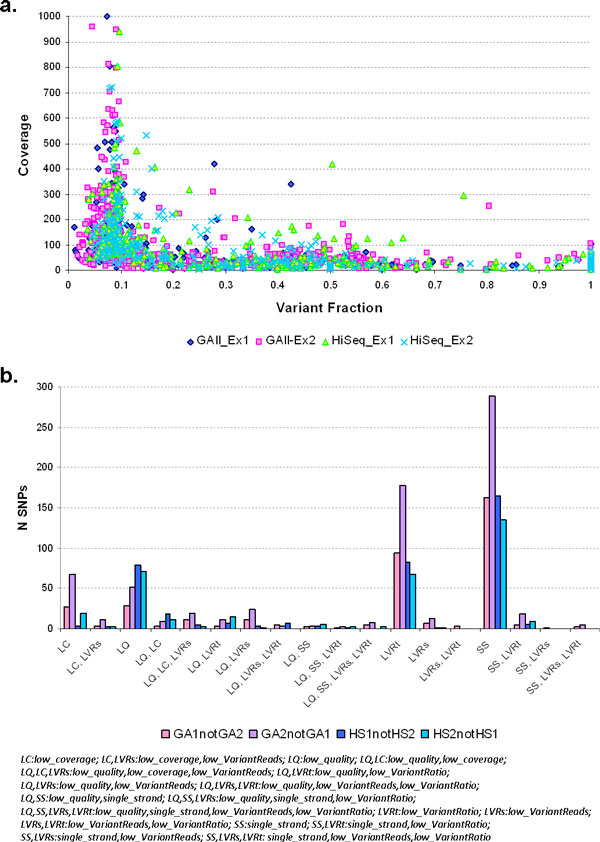
**Analysis of missed variants between Illumina ES runs**. (**a**) Distribution of variant fraction and coverage per base for missed SNPs in Illumina exome sequencing experiments. In the four Illumina exome sequencing runs, the variant fraction *vs*. coverage distribution of SNPs called in one run but not in the other three runs is as expected. The majority of single-run calls cluster in the lower range of variant fraction, below the standard threshold of 0.20-0.25 for reliable heterozygous calls. (**b**) Classification of filtered-out variants that differed between Illumina exome sequencing runs. Comparison of SNP variants called between exome sequencing runs using the same Illumina sequencing platforms (GAII *vs*. GAII and HiSeq1 *vs*. HiSeq2) shows that the most common parameter for differences between sequencing runs is the filtering-out of variants due to strand bias in one run versus another; the second parameter is the low variant ratio and third low quality of the called variants which in many cases is influenced by mis-mapping of reads to different other locations in the genome besides the specific target.

We also performed two additional exome sequencing experiments at lower depth of coverage using the latest Ion Technology (Life Technologies) using the Personal Genome Machine (PGM) and the Proton Machine. Comparison of all the exome experiments yielded a total of 21,772 concordant coding SNPs (cSNPs) across the four high-coverage Illumina exome sequencing experiments. Moreover, 18,623 high-quality cSNPs were concordant across the different platforms and across all six exome experiments, of which 8,777 are non-synonymous, including 73 nonsense changes. For the six different exome sequencing runs, the majority of single-run called SNPs fall below the 0.20-0.25 variant fraction for reliable heterozygous calls; those single-run calls with higher variant fractions (>0.20) generally fall below the 10x coverage cutoff (Figure 4). In the four Illumina exome sequencing runs, the variant fraction *vs*. coverage distribution of SNPs called in one run but not in the other three runs is as expected. The majority of single-run calls cluster in the lower range of the variant fraction distribution (Figure 5a). Comparison of SNP variants called between exome sequencing runs using the same Illumina sequencing platforms (GAII-1 *vs*. GAII-2 and HiSeq1 *vs*. HiSeq2) shows that the most common parameter for differences between sequencing runs is the filtering-out of variants due to strand bias; the second parameter is the low variant ratio, and thirdly the low quality of the called variants in one run *versus *another which in many cases is influenced by mis-mapping of reads to locations in the genome other than the specific target lowering the variant quality score (Figure 5b). The run that had the most 'private' calls was the PGM run; however the majority of these private calls fall within the low variant fraction portion of the distribution suggesting that most of them are false positives. Interestingly, the distribution of 'private' SNPs in the Proton exome sequencing run is more scattered and distributed in the coverage and variant fraction ranges usually observed for true positive variant calls (Figure 4). Of these high-quality cSNPs, 16,280 were recovered in the two WGS approaches in spite of the different platforms used and the differences in coverage between the two WGS experiments and among all the sequencing experiments.

Of note, comparison between the two WGS approaches, one being the original SOLiD WGS at approximately 30x average depth of coverage and the second one the Illumina HiSeq WGS at approximately 47x depth of coverage resulted in 3,090,120 concordant SNPs across the whole genome between the two experiments (90.34% of the original SOLiD WGS SNPs).

### Resolution of incidental findings from WGS

In most cases, the ES data recapitulated the WGS data: for example, both personal genome approaches identified 12 pharmacologically relevant variants (that is, pharmacogenetic traits) (Table 2). The greater coverage by ES (65 reads) *versus *WGS (2 reads) resolved the previously observed *CYP2C9 *p.Arg144Cys allele, involved in warfarin sensitivity, into a C/T heterozygous rather than a T/T homozygous allele. Similarly, the previously reported SMARD alleles were found, consistent with their identity as erroneous associations with disease in database entries. In addition to the *IGHMBP2 *allele reportedly associated with SMARD, three other 'disease-associated' variants found by WGS and confirmed as homozygous alleles and for which the proband did not manifest the disease (*GLB1*-chr3: 33,138,549G>A;p.P10L and *ABCA4*-chr1: 94,544,234T>C; p.H423R and *NHLRC1 *chr6: 18,122,506G>A; p.P111L, the latter allele resolved into a heterozygous variant by ES) have subsequently been observed as homozygous at a high frequency in public exome and genome sequence databases, again consistent with the original interpretation of erroneous entries as 'disease associated' in mutation databases.

**Table 2:** Observed pharmacogenetic variants by WGS *versus *ES.

Gene	OBS WGS	OBS WES	Variant	Drug(s)
*ADRB1*	C/C	C/C	p.Gly389Arg	Beta-blockers

*CDA*	A/C	A/C	p.Lys27Gln	Gemcitabine, cisplatin

*CYP2C8*	C/T	C/T	p.Arg139Lys	Paclitaxel

*CYP2C8*	T/C	T/C	p.Lys339Arg	Paclitaxel

*CYP2C9*	A/C	A/C	p.Ile359Leu	Warfarin

*CYP2C9*	T/T(r = 2)	C/T(r = 65)	p.Arg144Cys	Warfarin

*NAT2*	C/C	C/C	p.Ile114Thr	Isoniazid

*POR*	C/T	C/T	p.Ala503Val	Midazolam

*TPMT*	C/G	C/G	p.Ala80Pro	Purines

*UGT1A3*	C/C	C/C	p.Trp11Arg	Estrones, flavonoids

*UGT1A7*	C/C	C/C	p.Trp208Arg	Irinotecan

*UGT1A7*	G/G	G/G	p.Asn129Lys	Irinotecan

Elsewhere, the greater sensitivity of the ES data resolved false positive calls from the WGS. For example, the dominant *ABCD1 *ALD allele that had been previously reported was resolved as 'wild-type' (normal) with the further sequencing on the HiSeq platform. Examination of the individual sequence reads in the original WGS data showed that the available data had only six reads with three reference reads (two unique) and three variant reads (three unique). In contrast, the new ES data provided 232 reads at this base position. Thirty-five of these reads did contain the variant; however, the mapping algorithm (BWA) that was used to align the reads also showed all 35 were distinct, providing evidence for non-unique alignment. Hence, these 35 reads were more likely to represent other related genomic loci, such as a pseudogene or segmental duplications of the genomic interval containing this gene, and not the true *ABCD1 *locus.

**Figure 6:**
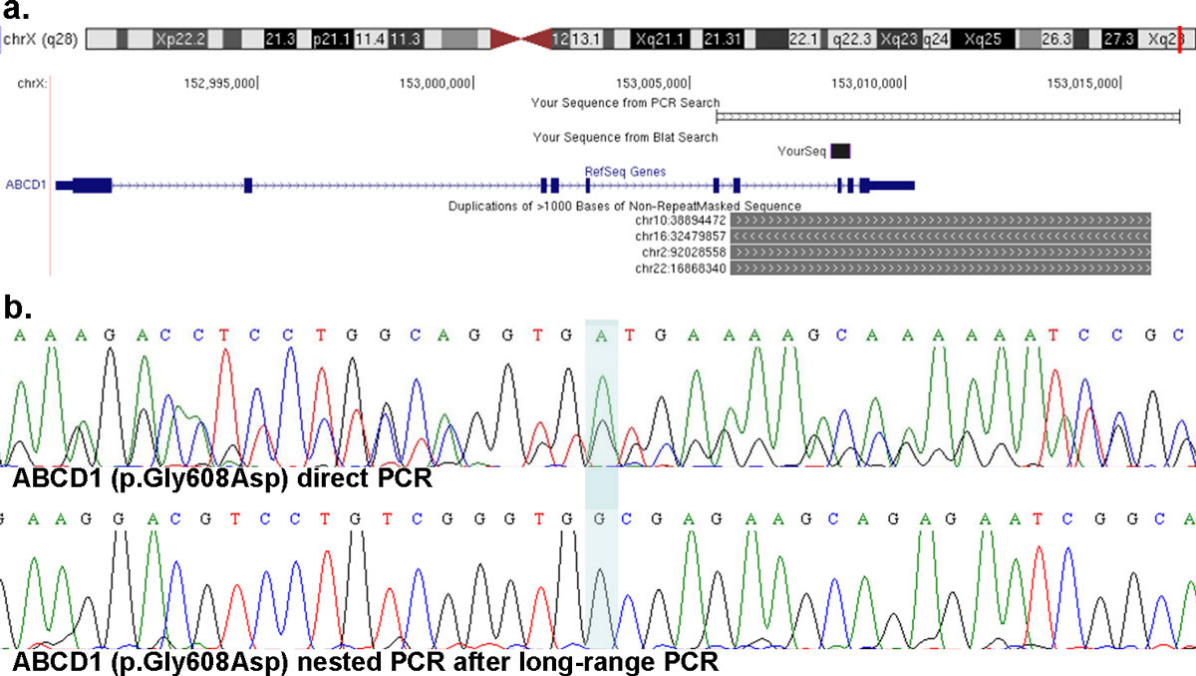
**(**a**) Genomic landscape of the region containing the *ABCD1 *gene and the repeated 9**.7 kb segment reported as a partial pseudogene. This segment comprises exons 7 to 10 of the *ABCD1 *gene and is repeated in four other locations in the reference genome with approximately 95% identity. (**b**) Comparison of Sanger sequencing of the reported mutation (p.Gly608Asp) through direct PCR of the segment containing exon 8 and 9 of *ABCD1 *using genomic DNA as a template (upper) and nested PCR of the same segment after long-range PCR amplification of an approximately 10 kb segment using primers specific to the *ABCD1 *locus outside of the repeated segment (lower).

Further data from PCR followed by either Sanger sequencing or *Hph*I restriction digestion, in all the family members, were not particularly helpful in resolving the true *ABCD1 *genotypes: each showed apparent 'heterozygosity' for this position (including the male subjects for this X-linked gene) (Figure 1), but were consistent with the model of cross-amplification due to genomic similarities between a distal segment of the *ABCD1 *gene and the presence in the personal genome of the proband of a partial pseudogene copy [20] of *ABCD1*. We observed that this particular region spanning 9,757 bp and comprising exons 7 through 10 of the *ABCD1 *gene is repeated four additional times in other locations in the haploid reference genome at chr2, chr10, chr16, and chr22 with 94.90% to 95.70% identity among the different copies (Figure 6a). It is difficult to experimentally assess by next-generation sequencing, hybridization, DNA specific endonuclease restriction digestion or computational mapping algorithms based on DNA sequence matching, to which of the duplicated copies of the region the reads that contain the presumed variant mismap. Our main interest was to test whether the functional *ABCD1 *gene contained the ALD mutation thus we designed primers for long-range PCR outside the duplicated segment that comprises exons 7 to 10 of *ABCD1 *to specifically interrogate the *ABCD1 *region. Long-range PCR amplification followed by nested regular PCR amplification of the exon 8 segment containing the variant and Sanger sequencing revealed that in fact the *ABCD1 *gene contained no mutation and that the variant allele is present in the pseudogene, although which of the four copies cannot be differentiated by this approach (Figure 6b). Hence, of all four approaches (WGS; ES; PCR+Sanger sequencing; PCR+restriction digestion), the observation that all the reads containing the variant had low mapping qualities in the high-coverage ES data and subsequent detailed analysis of the region to design a long-range PCR assay helped to interpret and give a faithful recapitulation of the published data [26], consistent with the X-linked hemizygosity expected in males. It seems that the ratio between the number of reads calling the reference base *versus *the number of mismapped reads calling variants can confound the variant calling algorithms. Copy number variant (CNV) alleles from pseudogenes, low copy repeats (LCRs), and other structural variants of personal genomes within clinical populations may confound the interpretation of simple nucleotide variant (SNV) alleles using whole genome approaches.

### Identification of a complex *SH3TC2 *allele associated with CMT

**Figure 7:**
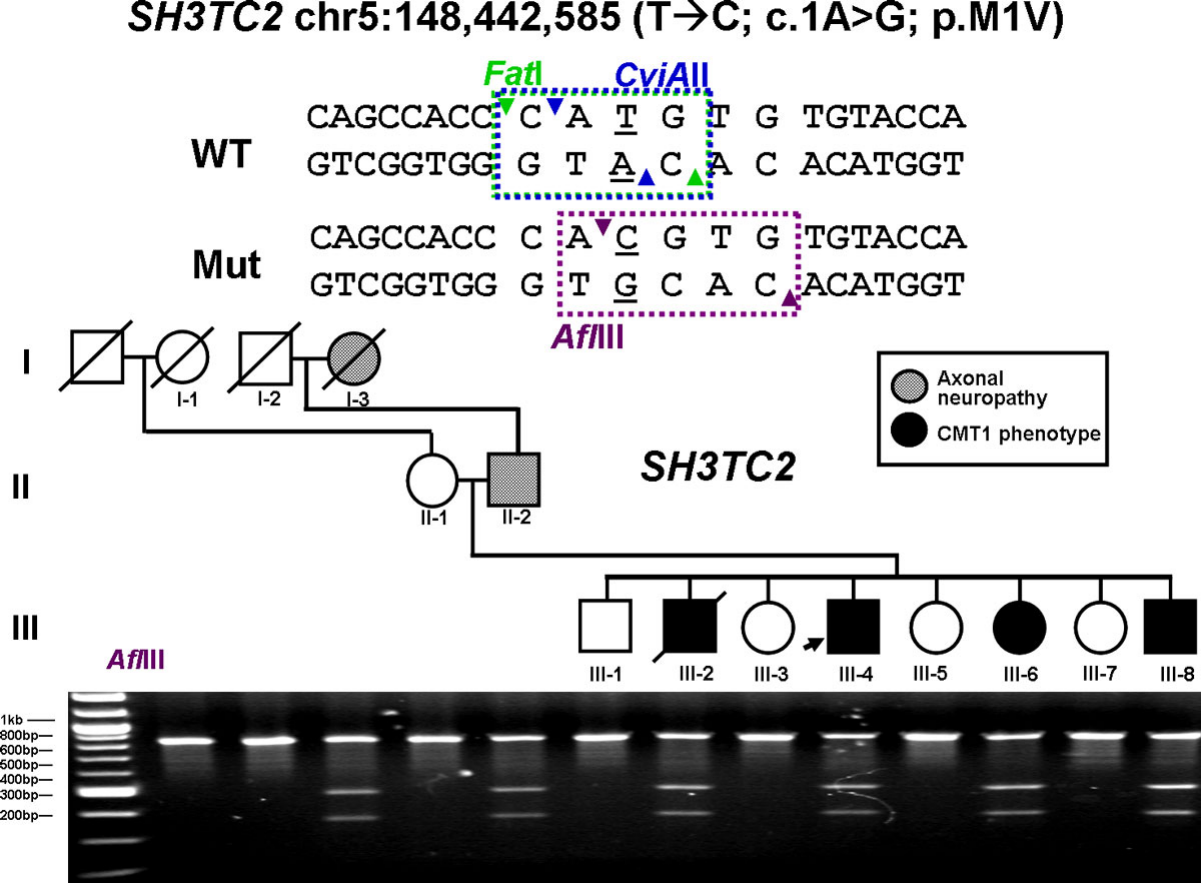
**Flanking sequence of the genomic mutation T → C at position chr5:148,422,778 in *SH3TC2***. The coding SNV transition c.1A>G causes a substitution of valine for methionine at the initiation codon. An alternative in frame ATG codon is present 90 codons downstream with a less conserved Kozak sequence than the upstream initiation codon. The wild-type allele can be recognized by two different restriction enzymes, *Fat*I and *CviA*II, but the mutation destroys the restriction site (data not shown). Conversely, transition T→C creates a restriction site that can be recognized by the endonuclease *Afl*III. The mutation segregates with the axonal neuropathy phenotype and co-segregates as a complex allele (p.M1?; Y169H) with the nonsense mutation p.R954X to cause the CMT phenotype in this family.

**Figure 8:**
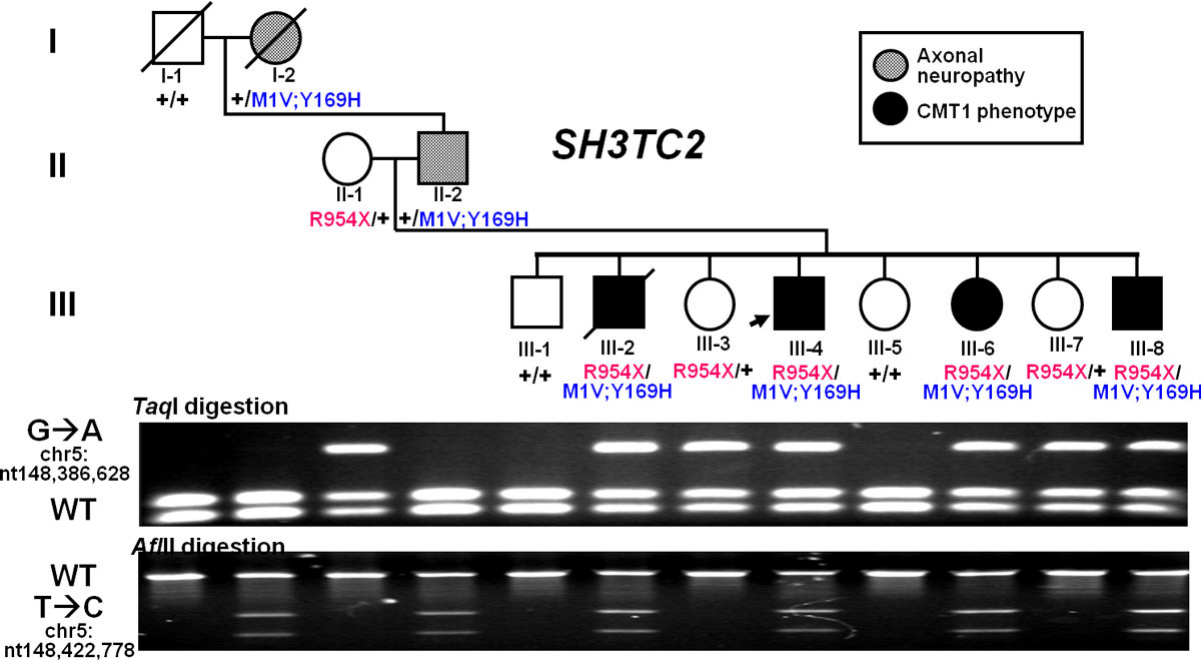
**Segregation of nonsense and complex alleles of *SH3TC2 *in a family with Charcot-Marie-Tooth neuropathy**. Previously reported nonsense variant (p.R954X) was inherited from the maternal line; reported missense p.Y169H and newly identified p.M1? variants are in *cis *inherited from the paternal line and segregating with the axonal neuropathy phenotype in the family. Individuals that inherited both the nonsense and complex allele present with CMT neuropathy.

**Figure 9:**
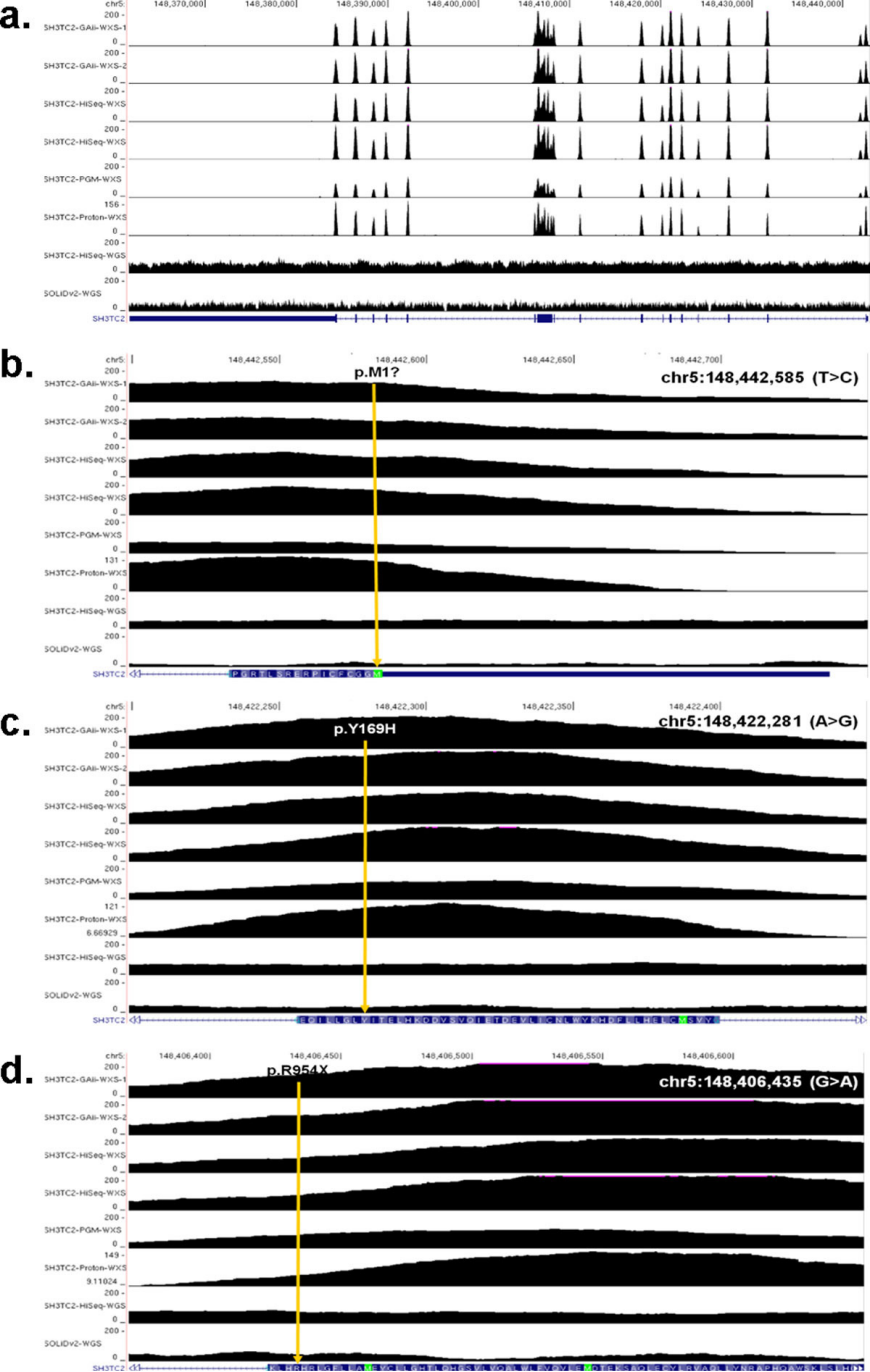
**Comparison of base pair coverage across the whole *SH3TC2 *gene and flanking regions of the three different mutations identified in the proband**. Exome sequencing (ES) provides saturation of base calling reads at >100x. (**a**) Coverage across the whole *SH3TC2 *gene. (**b**) Coverage across the p.M1? mutation. (**c**) Coverage across the p.Y169H mutation. (**d**) Coverage across the p.R954X mutation.

At the critical CMT causative locus in the proband, the *SH3TC2 *mutations (p.R954X and p.Y169H) that were identified by WGS were also found by ES. This was expected as these two sites had been extensively validated and studied in follow-up to the initial discovery. Surprisingly, however, an additional potentially significant mutation (p.M1?) was also found in the first codon of *SH3TC2 *by ES. At this position, the ES data contained 71 and 98 confidently mapping sequence reads corresponding to the reference base and variant base, respectively, consistent with heterozygosity at this position. The mutation was confirmed by PCR amplification followed by Sanger sequencing and three independent restriction endonuclease digestions (Figure 7) that showed the newly discovered allele segregating faithfully as a complex allele with the previously observed p.Y169H allele in the family (Figure 8). The three *SH3TC2 *gene variants were well-covered, depth of coverage ranging from 104x to 209x among the four different Illumina ES replicates, and identified or 'called' by the software in these four ES experiments with an approximate equal ratio for reference and variant reads over each position regardless of the platform used for sequencing. We also performed two additional ES experiments using the Personal Genome Machine (PGM) and Proton platforms from Life Technologies and an additional Illumina HiSeq whole-genome sequencing at 47.6 average depth of coverage to assess distribution of read depth in WGS using a different sequencing platform with longer reads. The three alleles in *SH3TC2 *were successfully covered and identified in these two additional ES and WGS approaches (Table 3, Figure 9).

**Table 3:** Comparison of *SH3TC2 *alleles in six exome sequencing experiments and one whole-genome sequencing.

Instrument		p.M1?	Ratio	p.Y169H	Ratio	p.R954x	Ratio
GAII (Ex1)	RR	47	0.48	90	0.50	63	0.47

	VR	66	0.58	90	0.50	71	0.53

	TR	113	-	180	-	134	-

GAII (Ex2)	RR	50	0.48	79	0.45	45	0.39

	VR	54	0.52	95	0.55	69	0.61

	TR	104	-	174	-	114	-

HiSeq (Ex1)	RR	71	0.42	100	0.48	54	0.47

	VR	98	0.58	109	0.52	61	0.53

	TR	169	-	209	-	115	-

HiSeq (Ex2)	RR	70	0.42	90	0.44	72	0.49

	VR	97	0.58	114	0.56	76	0.51

	TR	167	-	204	-	148	-

PGM (Ex)	RR	19	0.37	55	0.55	45	0.58

	VR	33	0.63	45	0.45	33	0.42

	TR	52	-	100	-	78	-

Proton (Ex)	RR	58	0.55	79	0.50	35	0.56

	VR	48	0.45	80	0.50	28	0.44

	TR	108	-	159	-	63	-

HiSeq-WGS	RR	29	0.60	25	0.43	32	0.48

	VR	19	0.40	33	0.57	34	0.52

	TR	48	-	58	-	66	-

RR: number of reads calling the reference base; TR: total number of reads at that position; VR: number of reads calling the variant base. The ratio columns reflect the fraction of reads that called either the reference base or the read base over the total number of reads for that position.

Retrospective analysis of the prior WGS SOLiD data [2] revealed that the total accumulated DNA sequence reads covering this site had satisfied the applied filters (total of 15 unique sequencing reads covering position chr5:148,442,585). The fraction of those reads, however, that represented the variant allele was below the accepted threshold (two reads of the total 15, 13.3% *versus *the filter's cutoff >20% variant allele fraction). Hence, the site was adequately covered overall, but not by enough reads containing the variant allele for the bioinformatic algorithm to call it a variable position. Others have suggested an intrinsic allelic bias in the ligation based SOLiD sequencing method, however, this individual example can be readily explained stochastically [27,28].

These new data regarding *SH3TC2 *variant alleles suggest three possibilities: (1) the newly identified p.M1? variant is the causative allele in *cis *with a benign p.Y169H; (2) the p.M1? variant alone has little effect because of possible re-initiation of translation from an alternative methionine initiation codon 90 amino acids downstream and hence pathogenicity of this allele results from the downstream p.Y169H; or (3) the CMT causality requires the combination of variants in the complex allele (p.M1?; p.Y169H) plus the co-segregating nonsense allele p.R954X [29]. This latter hypothesis would be consistent with a 'mutational load' model, in which a partial loss-of-function hypomorphic complex allele is independently segregating with the electrophysiologically identified axonopathy as observed for the homozygous *PMP22 *T118M mutation [30]. Rare variant alleles and combinations of such alleles at a locus may aggregate from parental contributions or may occur by new mutations within recent ancestors; a concept referred to as clan genomics [31]. It is distinctly possible that the p.M1? variant occurred *de novo *on a haplotype that contains p.Y169H within the clan. Of note, while the p.M1? variant is novel, since our original report [2] the p.Y169H variant was observed in additional individuals. However, it is always observed in the heterozygous state and never as a homozygous variant, making its functional significance elusive.

Western blotting of lymphoblastoid cell lines derived from the subject and family members carrying different variant alleles at the *SH3TC2 *locus identified, using a specific anti-*SH3TC2 *antibody, only one smaller band of a size consistent with the protein product of a predicted spliced variant of *SH3TC2 *(Additional File 1). Thus, in these lymphoblastoid cell lines from the subject and other family members segregating the alleles, no evidence of a truncated protein, perhaps reflecting nonsense mediated decay of the nonsense mutation bearing transcript, nor evidence of a predicted sized protein due to re-initiation of translation from an internal AUG initiation codon could be obtained.

These new findings from ES studies further support the original study conclusions of: (1) pathogenic involvement of the *SH3TC2 *gene; and (2) spurious interpretations of 'incidental' findings that may occur based upon current database entries. Further, these new data illuminate how structural variation unique to individuals from clinical populations might challenge interpretation of some variant alleles.

## Conclusions

These data illustrate that, as predicted by an editorial accompanying the original manuscript [32], ES can indeed identify the causative variants of a mendelian genetic disease. In many instances, the high yet skewed depth of coverage in targeted regions afforded by the ES methods may offer higher likelihood of recovery of significant variants and resolution of their true genotypes when compared to the lower, but more uniform WGS coverage (Table 4). This may be particularly true because most WGS studies are typically implemented at average depths of coverage well below any current ES approach, due to the theoretical assumption and observations that an approximately 30x average depth of coverage across the whole genome would provide enough read depth to identify >99% of the homozygous variants and approximately 98% of the heterozygous variants; that is, NOT identifying 1% to 2% of variants in the coding regions. The ability to detect rare variant alleles may be particularly relevant to medically actionable variants [31] and pharmacogenetic traits [33]; both providing personal genome variation of potential utility in precision medicine. Given the high coverage at which targeted exome sequencing is performed (>100x) and the reduced sampling space provided by the capture step, the percentage of targeted bases that are not sequenced is low due to the distribution of individual reads throughout the 'genome space' interrogated and the fold sequence coverage for each base position. However, the technical limitation of ES might be imposed by the capture step [34], due to limitations of the capture designs (that is, not all genes and exons have been adequately defined and therefore not included in ES designs) and the capture efficiency (70% to 80%). Our data suggest that the choice of ES for identification of fully penetrant critical SNV mutations in the coding regions of mammalian genomes may be regarded as superior, rather than a shortcut, or compromise, when compared with WGS approaches at depths of coverage below 100x. WGS continues to be a more comprehensive next-generation sequencing approach to total variant detection in a personal genome if one seeks to capture the majority of the genome-wide variation including CNVs and other structural variants in addition to SNVs. However, in order to best capture variation higher coverage at lower costs need to be implemented for WGS approaches.

**Table 4:** Variant call comparison across all platforms for variants mentioned throughout the text.

Gene	chr	pos_hg19	Ref	var	aa_change	SOLiD-WGS	GAII-Ex1	GAII-Ex2	HiSeq-Ex1	HiSeq-Ex2	PGM-Ex	Proton-Ex	HiSeq-WGS
*ABCD1*	chrX	153,008,483	G	A	G608D	het (3:3)	het (49:183)	het (35:197)	N/A	N/A	N/A	N/A	het (8:20)

*IGHMPB2*	chr11	68,705,674	C	A	T879K	hom (9:1)	hom (7:90)	hom (107:1)	hom (147:0)	hom (115:0)	hom (52:0)	hom (113:0)	hom (52:0)

*GLB1*	chr3	33,138,549	G	A	P10L	hom (14:0)	hom (139:0)	hom (163:0)	hom (254:1)	hom (279:1)	hom (68:0)	hom (147:0)	N/A

*ABCA4*	chr1	94,544,234	T	C	H423R	hom (13:1)	hom (170:0)	hom (131:0)	hom (148:0)	hom (137:3)	hom (89:0)	hom (84:1)	hom (50:0)

*NHLRC1*	chr6	18,122,506	G	A	P111L	hom (3:0)	het (9:6)	N/A	het (9:14)	het (9:11)	N/A	N/A	het (16:17)

*SH3TC2*	chr5	148,442,585	T	C	M1	N/A	het (47:66)	het (50:54)	het (71:98)	het (70:97)	het (19:33)	het (46:80)	het (29:19)

*SH3TC2*	chr5	148,422,281	A	G	Y169H	het (17:13)	het (90:90)	het (79:95)	het (100:109)	het (90:114)	het (55:45)	het (67:63)	het (25:33)

*SH3TC2*	chr5	148,406,435	G	A	R954X	het (20:15)	het (63:71)	het (45:69)	het (54:61)	het (72:76)	het (45:33)	het (37:33)	het (32:34)

## Competing interests

JRL is a paid consultant for Athena Diagnostics, holds stock ownership in 23andMe and Ion Torrent Systems, and is a co-inventor on US and European patents related to molecular diagnostics. RAG is a paid consultant of G.E. Clarient. The Department of Molecular and Human Genetics at Baylor College of Medicine derives revenue from genetic testing offered in the Whole Genome Laboratory (WGL) and Medical Genetics Laboratories (MGL: http://www.bcm.edu/geneticlabs). The remaining authors declare that they have no competing interests.

## Authors' contributions

JRL and RAG conceived and designed the study and participated in the drafting and writing of the manuscript. CG-J performed data analyses, variant confirmations and analyses, and contributed to the writing of the manuscript. YY, MNB, and SJ performed data analysis. CJB, CLK, MW, and ACH performed data generation and analysis and helped with the writing of the methods. CE and JGR participated in the coordination of the study. DMM participated in the coordination of the study, data generation, and writing of the manuscript. All authors read and approved the final manuscript.
